# Genome‐based prediction of multiple wheat quality traits in multiple years

**DOI:** 10.1002/tpg2.20034

**Published:** 2020-07-25

**Authors:** Maria Itria Ibba, Jose Crossa, Osval A. Montesinos‐López, Abelardo Montesinos‐López, Philomin Juliana, Carlos Guzman, Emily Delorean, Susanne Dreisigacker, Jesse Poland

**Affiliations:** ^1^ International Maize and Wheat Improvement Center (CIMMYT) Km 45 Carretera Mexico‐Veracruz CP 52640 Mexico; ^2^ Colegio de Postgraduados (COLPOS), Montecillos Edo. de México CP 56230 México; ^3^ Facultad de Telemática Universidad de Colima Colima México; ^4^ Departamento de Matemáticas Centro Universitario de Ciencias Exactas e Ingenierías (CUCEI), Universidad de Guadalajara Guadalajara Jalisco 44430 México; ^5^ Departamento de Genética Escuela Técnica Superior de Ingeniería Agronómica y de Montes, Campus de Rabanales, Universidad de Córdoba Córdoba Spain; ^6^ Department of Agronomy Kansas State University, 2004 Throckmorton Plant Science Center Manhattan KS 66506 USA

## Abstract

Wheat quality improvement is an important objective in all wheat breeding programs. However, due to the cost, time and quantity of seed required, wheat quality is typically analyzed only in the last stages of the breeding cycle on a limited number of samples. The use of genomic prediction could greatly help to select for wheat quality more efficiently by reducing the cost and time required for this analysis. Here were evaluated the prediction performances of 13 wheat quality traits under two multi‐trait models (Bayesian multi‐trait multi‐environment [BMTME] and multi‐trait ridge regression [MTR]) using five data sets of wheat lines evaluated in the field during two consecutive years. Lines in the second year (testing) were predicted using the quality information obtained in the first year (training). For most quality traits were found moderate to high prediction accuracies, suggesting that the use of genomic selection could be feasible. The best predictions were obtained with the BMTME model in all traits and the worst with the MTR model. The best predictions with the BMTME model under the mean arctangent absolute percentage error (MAAPE) were for test weight across the five data sets, whereas the worst predictions were for the alveograph trait ALVPL. In contrast, under Pearson's correlation, the best predictions depended on the data set. The results obtained suggest that the BMTME model should be preferred for multi‐trait prediction analyses. This model allows to obtain not only the correlation among traits, but also the correlation among environments, helping to increase the prediction accuracy.

**Abbreviations**
ALV: average abscissa of rupture
ALVP: alveograph maximum overpressure
ALVPL: curve configuration ratio
ALVW: dough deformation energy
APC: average Pearson's correlation
BMTME: Bayesian multi‐trait multi‐environment
EYT: elite yield trial
FLRPRO: flour protein
FLRSDS: sodium dodecyl sulfate flour sedimentation volume
GBS: genotyping‐by‐sequencing
GRNHRD: grain hardness
GRNPRO: grain protein
GS: genomic selection
LOFVOL: bread loaf volume
MAAPE: mean arctangent absolute percentage error
MIXTORQ: mixograph torque
MIXTIM: mixograph peak time
MTR: multi‐trait ridge regression
NIRS: near‐infrared reflectance spectroscopy
SKCS: single‐kernel characterization system
SNP: single nucleotide polymorphism
TESTWT: test weight
TKW: 1000‐kernel weight
YT: first year yield trial.

## 1 INTRODUCTION

Wheat, the third most cultivated crop in the world, is one of the major sources of energy and proteins in the human diet in both developed and developing countries. Its importance is undoubtedly related to the unique viscoelastic properties of wheat dough, which allows utilizing wheat to produce a plethora of food products such as different types of bread, pizza, pasta and cookies. However, in order to produce all these products, specific quality requirements are needed in terms of protein quantity, quality and kernel hardness (Peña, Trethowan, Pfeiffer, & van Ginkel, 2002).

In recent years, wheat consumption has grown continuously, especially in countries undergoing urbanization and industrialization. These factors, together with globalization, have led to a gradual shift from household production to the industrial production of wheat‐based foods (Shewry et al., 2015). With this shift, the importance of wheat quality also increased, since at the industrial level, stricter and uniform processing and end‐use quality properties are required. However, in the context of a breeding program, the analysis of wheat quality has several limitations because the tests required are typically expensive, time‐consuming and require a relatively large amount of seeds. For these reasons, screening for wheat quality does not typically happen until late in the breeding cycle, with the result that wheat lines with undesirable quality characteristics are often advanced (Battenfield et al., 2016).

Genomic selection (GS) applied at different stages of the breeding cycle could greatly facilitate the selection accuracy for wheat quality, improve the overall quality of advanced wheat lines and considerably reduce the cost of the screening process. Briefly, GS consists of using the phenotypic and genotypic data of a training population in order to predict the phenotypic values of a testing population that has only been genotyped. The two main factors affecting the efficiency of genomic selection are the heritability of the phenotypes to be predicted and the choice of the training population in terms of size, diversity and relationship with the testing population (Crossa et al., 2017).

In general, the heritability of a trait depends on the proportion of that trait which is not determined by the environment. Wheat quality traits mostly exhibit moderate to high narrow‐sense heritability (*h^2^*) values. For example, gluten strength, as indicated by the alveograph deformation energy (W), exhibited *h^2^* values ranging from 0.65 to 0.72 (Battenfield et al., 2016; Kristensen et al., 2019), whereas *h^2^* values ranging from 0.51 to 0.60 were identified for dough extensibility (Hayes et al., 2017; Kristensen et al., 2019). In general, both grain and protein content proved to be less heritable, with *h^2^* values between 0.17 and 0.57 (Battenfield et al., 2016; Hayes et al., 2017), whereas traits such as flour yellowness appeared to be almost completely genetically controlled (*h^2^* = 0.93, Hayes et al., 2017). Despite being relatively highly heritable, however, most quality traits are polygenic (Jernigan et al., 2018), thus complicating the use of marker‐assisted selection for their routine analysis and representing, in contrast, an ideal target for GS.

Another factor that influences the accuracy of GS is the training population. In general, the size of the training population depends on the relationship between the training and testing population: the more genetically distant the two populations are, the bigger the size of the training population required to obtain accurate predictions. Also, the use of a diverse training population typically increases the reliability of the predictions (Edwards et al., 2019). The importance of the training population for accurately predicting wheat quality traits became apparent in the study of Battenfield et al. (2016), where greater prediction accuracy was gradually achieved by increasing the size of the training population over the years (from 250 to 4095 lines). However, obtaining a large training population that has been characterized for multi‐wheat quality traits is not easy to achieve due to the cost of the analyses and the time and seed quantity required to perform them. For these reasons, only a few breeding programs have the capacity (and the data) necessary to build solid genomic prediction models for wheat quality.

Regarding genomic prediction models, researchers have found that the genetic correlation between multi‐traits is the basis for the benefit of a multivariate analysis, because the greater the relatedness between traits, the greater the benefit of multivariate analysis. The advantage of using a multivariate analysis to estimate covariance parameters is that it increases the precision of the parameter estimates because it takes into account the genetic (and residual) correlation between the traits and environments under study (Montesinos‐López et al., 2016, 2019a, 2019b). Although Bayesian multivariate models are computationally intensive, they are very effective in providing parsimonious and informative analyses. The Bayesian multi‐trait multi‐environment models proposed by Montesinos‐López et al. (2016) allows increasing prediction accuracy and parameter estimates resulting in unbiased estimates of secondary traits selected under indirect selection. Multivariate analysis also allows predicting a trait when genotypes have been measured for other traits.

Algorithms have been developed for fitting generalized linear models including regression, logistic regression, and multinomial regression problems (Friedman, Hastie, & Tibshirani, 2010). These algorithms work well on large genomic data sets and are implemented in an R package named *glmnet*. The *glmnet* allows implementing the Multi‐Trait Ridge regression (MTR) model for a Gaussian response variable that contains a quadratic (ridge) penalty on the coefficients for each variable. The MTR model was implemented with the Lasso and Elastic‐Net Regularized Generalized Linear Models (*glmnet*) R package (Friedman et al., 2010).

At the International Maize and Wheat Improvement Center (CIMMYT), every year ∼1400 lines from the spring bread wheat preliminary or first year yield trial (YT) are fully characterized for the quality of their processing traits. The information generated is then used by the breeders to advance the highest yielding lines with the best quality to the next cycle. The selected lines (∼600) are then analyzed a second year for their quality characteristics and undergo a second selection by the breeders (Guzmán et al., 2016). The wheat quality multi‐trait information generated every year can be used to develop solid prediction models that could help reduce or fine‐tune the selection of wheat lines for the second year quality analysis. These models could greatly help reduce the cost of the wheat quality analysis and its efficiency by discarding lines that are predicted to have undesirable quality and analyzing only the lines that are promising for their processing characteristics.

In the present study, the quality data developed for the ∼1400 lines of the YT have been used to develop a forward prediction model to estimate the quality characteristics of the lines selected for the second year quality analysis. The accuracy of the prediction model was then estimated across five cycles in order to determine the efficiency of introducing such a method for routine selection in a wheat breeding program.

**Core Ideas**

- Wheat quality traits can be predicted with high to moderate prediction accuracies
- Genomic selection could be implemented to improve selection for wheat quality
- The Bayesian multi‐trait multi‐environment model should be preferred for prediction

## 2 MATERIALS AND METHODS

### 2.1 Plant material

In the present study, the common spring wheat lines selected for the quality analysis from the first year yield trial (YT), were used as the training population to predict the quality of the wheat lines selected from the elite yield trial (EYT) for a second year quality analysis. The analysis was performed using five sets of data as reported below:

‐ **Data 1** made up of 1345 lines from the 2013–2014 YT and 658 lines from the 2014–2015 EYT trial.
‐ **Data 2** made up of 1360 lines from the 2014–2015 YT and 686 lines from the 2015–2016 EYT trial.
‐ **Data 3** made up of 1384 lines from the 2015–2016 YT and 622 lines from the 2016–2017 EYT trial.
‐ **Data 4** made up of 1447 lines from the 2016–2017 YT and 614 lines from the 2017–2018 EYT trial.
‐ **Data 5** made up of 1522 lines from the 2017–2018 YT and 556 lines from the 2018–2019 EYT trial.

All the plant material was grown at the Campo Experimental Norman E. Borlaug in Ciudad Obregon, Sonora, Mexico, under full irrigation and planted following a lattice design.

### 2.2 Phenotypic data

All quality analyses were performed according to the AACCI International approved methods or other modified methods described in Battenfield et al. (2016).

Specifically, 1000‐kernel weight (TKW, g) was estimated using the digital image system SeedCount SC5000 (Next Instruments) for samples from years 2013–2014 to 2015–2016. However, starting from years 2016–2017, TKW was calculated using the Single‐Kernel Characterization System (SKCS, Perten Instruments, Sweden). Test weight (TESTWT, kg hl^−1^) was measured using a 37.81‐ml sample. Grain protein (GRNPRO) and moisture content were determined by Near‐Infrared Reflectance Spectroscopy (NIRS) according to the official methods AACC 39‐10 and 39‐00, respectively (AACC, 2010). The GRNPRO was reported at 12.5% moisture.

The grain hardness (GRNHRD) of the samples from years 2013–2014 to 2015–2016 was estimated through NIR calibrated according to particle size index values following the AACC method 39‐70A (AACC, 2010). Starting from the year 2016–2017, the hardness of the grain samples was analyzed using the SKCS following the AACC method 55‐31 (AACC, 2010). Depending on grain hardness and moisture content, grain samples were optimally tempered to 13% (soft wheat) or 16.5% (hard wheat), and milled using a Brabender Quadrumat Senior mill (C. W. Brabender OHG).

The flour protein (FLRPRO) and moisture content were then estimated by NIR calibrated according to AACC Method 46‐11A and AACC Method 44‐15A, respectively (AACC, 2010). The FLRPRO was reported at 14% moisture content. Sodium dodecyl sulfate (SDS) sedimentation (FLRSDS) was conducted as in Peña, Amaya, Rajaram, and Mujeeb‐Kazi (1990). A 35 g mixograph was used to estimate the dough mixing and rheological properties, following the AACC method 54‐40A (AACC, 2010). The following parameters were recorded: time to peak mixing strength (MIXTIM) and height at the midline of peak mixing strength (MIXTORQ). Dough rheological characteristics were also assessed using the Chopin Alveograph (Tripette & Renaud) with a 60 g flour sample and according to the AACC method 54‐30A (AACC, 2010). For this analysis, the following parameters were recorded: dough deformation energy, indicative of the overall gluten strength (ALVW); maximum overpressure, indicative of dough tenacity (ALVP); average abscissa of rupture, indicative of dough extensibility (ALVL); and curve configuration ratio, indicative of the ratio between dough tenacity and extensibility (ALVPL). Both the mixograph and alveograph methods were adjusted for unified optimum water absorption based on solvent retention capacity, as reported by Guzmán, Posadas‐Romano, Hernández‐Espinosa, Morales‐Dorantes, and Peña (2015). The lines were also assessed for yeast‐leavened bread quality following the AACC method 10‐09 (AACC, 2010) and using the Guzmán et al. (2015) adjustment for optimal water absorption. Bread loaf volume (LOFVOL) was measured by rapeseed displacement in accordance with AACC method 10‐05.01 (AACC, 2010). Correlation among the phenotypes was calculated using SAS University Edition (SAS/STAT, SAS Institute Inc, NC, USA).

### 2.3 Genotypic data

All the lines were genotyped using the genotyping‐by‐sequencing approach (Poland, Brown, Sorrells, & Jannink, 2012) at Kansas State University. The TASSEL v.5 (Trait Analysis by Association Evolution and Linkage) GBS pipeline was used to call marker polymorphisms (Glaubitz et al., 2014), and a minor allele frequency of 0.01 was used for single nucleotide polymorphism (SNP) discovery. The resulting 6,075,743 unique tags were aligned to the bread wheat genome reference sequence (RefSeq v.1.0) (IWGSC 2018) with an alignment rate of 63.98%. After filtering for SNPs with an inbred coefficient > 80%, p‐value for Fisher's exact test < .001 and χ^2^ value lower than the critical value of 9.2, we obtained 78,606 GBS markers that passed at least one of those filters. These markers were further filtered for less than 50% missing data, greater than 0.05 minor allele frequency and less than 5% heterozygosity, in all the datasets. Markers missing data were imputed using the ‘expectation‐maximization’ algorithm in the ‘R’ package rrBLUP (Endelman, 2011).

### 2.4 Genome‐based statistical models

Two multivariate models were fitted. The first model is the Bayesian Multi‐Trait Multi‐Environment (BMTME) proposed by Montesinos‐López et al. (2016) and the second model used in this study is the multi‐trait ridge regression (MTR) model (Friedman et al., 2010).

#### 2.4.1 Bayesian multi‐trait multi‐environment (BMTME)

The first model is the Bayesian Multi‐Trait Multi‐Environment (BMTME) proposed by Montesinos‐López et al. (2016):

(1) $$Y=X_{E}B+Z_{1}b_{1}+Z_{2}b_{2}+e$$

where **Y** is the phenotypic response matrix of order n×L, where each column corresponds to a trait and *L* is the number of traits, $X_{E}$ is design matrix for the environment effects of order n×I, **B** is a matrix of beta coefficients of order I×L, **Z**_1_ is the design matrix of genotypes of order n×J, and **Z**_2_ is the design matrix of genotype × environment interaction of order n×JI; **b**_1_ is the matrix of genotypic random effects of order J×L that we assume is distributed as $b_{1}∼MN\left(0,G,\Sigma_{t}\right)$ where MN stands for matrix normal distribution with mean vector **0** and within and between variance‐covariance matrices **G** and $\Sigma_{t}$, respectively. **G** is calculated as suggested by Van Raden (2008) and assumed known. $\Sigma_{t}$ is an unknown variance‐covariance matrix of traits of dimension L×L; **b**_2_ is the matrix of genotype × environment interaction random effects of order JI×L and we assume that it is distributed as $b_{2}∼MN\left(0,\Sigma_{E}⊗G,\Sigma_{t}\right)$; **e** is a random matrix of errors of order n×L distributed as $MN(0,I_{n},R_{e})$ (Montesinos‐López et al., 2019a, 2019b).

#### 2.4.2 The prior distributions of the BMTME

The complete Bayesian specification of this model assumes independent multivariate normal distributions for the columns of **B**, that is, for the fixed effect of each trait a prior multivariate normal distribution is adopted, $\beta_{t}∼MN\left(\beta_{t0},\Sigma_{\beta_{t}}\right);$ and for the variance‐covariance components of beta coefficients ($\Sigma_{\beta_{t}}),$ traits ($\Sigma_{t}),\;$environments ($\Sigma_{E}$) and Residual ($R_{e}$) were used inverse‐Wishart (*IW*) distributions.

The hyper‐parameters for this variance‐covariance components were obtained partitioning the total variance‐covariance of the phenotypes into two components: (1) the error and (2) the linear predictor and from this we ended up with the following hyper‐parameters (for details, see Montesinos‐López, Montesinos‐López, Gianola, Crossa, & Hernández‐Suárez, 2018 [Appendix A]). For this reason, for $\Sigma_{\beta_{t}}∼IW\left(df_{\beta t},S_{\beta t}\right)$ with $df_{\beta t}=L+1$, $S_{\beta t}$ = $\frac{R_{1}^{2}V_{y}\;\times \;\left(df_{\beta t}+L+1\right)}{MS_{\beta t}}$ ; $V_{y}$ is the computed phenotypic variance‐covariance matrix, $MS_{\beta t}=\sum_{i=1}^{n}x_{i}^{T}x_{i}/n$. $R_{1}^{2}=0.25$ is the proportion of variance‐covariance that is assumed is explained *a priori* by the traits in the fixed effects. For $\Sigma_{T}∼IW\left(df_{t},S_{t}\right)$, with $df_{t}=L+1$, $S_{t}=\frac{R_{2}^{2}V_{y}\;\times \;\left(df_{t}+L+1\right)}{MS_{b1}}+\frac{R_{3}^{2}V_{y}\;\times \;\left(df_{t}+L+1\right)}{MS_{b2}}$, $R_{2}^{2}=R_{3}^{2}=0.25$, are the proportion of variance‐covariance that *a priori* is explained by the traits in the genotype × trait and genotype × environment × trait interaction terms; $MS_{b1}=\left(\sum_{i=1}^{n}z_{1i}^{T}G_{g}z_{1i}\right)/n$ and $MS_{b2}=\left(\sum_{i=1}^{n}z_{2i}^{T}\left(\Sigma_{E}⊗G_{g}\right)z_{2i}\right)/n$. For $\Sigma_{E}∼IW\left(df_{E},S_{E}\right)$ with $df_{E}>I+1$, with I=2, $S_{E}=\frac{R_{3}^{2}V_{y*}\;\times \;\left(df_{E}+I+1\right)}{MS_{b2*}}$ and with $MS_{b2*}=tr\left(\frac{S_{t}}{df_{t}+L+1}⊗G\right)$. For $R_{e}∼IW\left(df_{e},S_{e}\right)$ with $df_{e}>L+1$ and $S_{e}=\left(1-R_{1}^{2}-R_{2}^{2}-R_{3}^{2}\right)V_{y}\times \left(df_{e}+L+1\right)$ where *L* denotes the number of traits for this reason we used 11 and 13 since in some data sets we have 11 and in the other 13 traits.

#### 2.4.3 Multi‐trait ridge regression (MTR)

The second multivariate model used in this study is the multi‐trait ridge regression (MTR) model for Gaussian response variables that contains a quadratic (ridge) penalty on the coefficients for each variable (Friedman et al., 2010). The method minimized the following loss function:

$$\hat{B}\left(\lambda \right)=\underset{B}{\mathrm{argmin}}{\left|\left|Y-1_{n}\beta_{0}^{T}-\mathrm{XB}\right|\right|}^{2}+\sum_{t=1}^{n_{T}}\lambda_{t}{\left|\left|\beta_{t}\right|\right|}^{2}$$

$$\underset{B}{=\mathrm{argmin}}{\left|\left|Y-1_{n}\beta_{0}^{T}-XB\right|\right|}^{2}+\mathrm{tr}\left[\mathrm{Diag}\left(\lambda \right)B^{T}B\right]$$

where $\beta_{0}={\left(\beta_{1},\;\ldots ,\;\beta_{L}\right)}^{T}$ is the vector with the intercepts for each trait. As input variables ($X=\{x_{ip}\},$ *i = 1,2,..,n; p = 1,2,..*,$\;N_{1}$) for the proposed MTR model, we concatenated the information of environments, the information of markers through the Cholesky decomposition of the genomic relationship matrix and the information of the genotype × environment (G×E) interaction.

Due to the above reasons, we built the design matrices of environments ($Z_{E})$, genotypes ($Z_{G})$ and G×E ($Z_{GE});$ then we obtained the Cholesky decomposition of the genomic relationship matrix (**G**). Then we post‐multiplied the design matrix of genotypes by the transpose of the upper triangular factor of the Cholesky decomposition ($Q^{T}$), $Z_{G}^{*}=Z_{G}Q^{T}$, and finally the G×E term was obtained as the product of the design matrix of the G×E term post‐multiplied by the Kronecker product of the identity matrix that is equal to the number of environments and $Q^{T}$, that is, $\;Z_{GE}^{*}=Z_{GE}(I_{I}⊗Q^{T}$). Finally, the matrix with input covariates used for implementing the multi‐trait ridge regression model was equal to $X=[Z_{E},Z_{G}^{*},\;Z_{GE}^{*}]$. $B=[\beta_{1},\;\ldots ,\;\beta_{L}]$ is the matrix with the coefficient effects and each column corresponds to each trait, $\beta_{t}={\left(\beta_{t1},\;\ldots ,\;\;\;\beta_{tp}\right)}^{T}$, t=1,…,L, and $\lambda ={\left(\lambda_{1},\;\ldots ,\lambda_{L}\right)}^{T}$ is the vector containing the regularization parameters of all the traits.

To estimate the hyperparameter λ for each outer training set, we implemented a 10‐fold inner cross‐validation evaluating 100 values of lambda and used them as metrics for selecting the optimal hyperparameter (λ), the mean square error of prediction. Then with the optimal λ the model was refitted with the whole outer training set and the trained model was used to predict the outer testing set. This MTR model was implemented with the Lasso and Elastic‐Net Regularized Generalized Linear Models (*glmnet)* R package (Friedman et al., 2010).

### 2.5 Evaluation of genomic‐enabled prediction accuracy

Evaluation of the genomic‐enabled prediction accuracy was performed with cross‐validation in order to avoid using the same data set for training and testing and thus obtaining overoptimistic predictions. There are several strategies that can be used for cross‐validation, but here we used one that mimics the sparse testing conducted in plant breeding programs. The data used in this study comprise: (1) Data 1, which has lines evaluated in year 2013–2014 as the training set to predict most individuals observed in the field in year 2014–2015; (2) Data 2, which comprises lines from year 2014–2015 as training used to predict the majority of lines evaluated in year 2015–2016; (3) Data 3, which included lines evaluated in year 2015–2016 to predict the majority of lines evaluated in 2016–2017; (4) Data 4, which had lines evaluated in 2016–2017 and used as training to predict most of the lines evaluated in 2017–2018; and (5) Data 5, which included lines tested in 2017–2018, which were used to predict most of the lines of 2018–2019.

Since we had the same lines evaluated in two consecutive years, we used as the testing set 90% of the lines of the second year and as the training set the remaining information (full information of the first year plus 10% of the lines in the second year). We repeated this sampling process five times and each time the samples of the 90% (for testing) and 10% (for training) were obtained randomly. Finally, we reported as prediction accuracy Pearson's correlation and the mean arctangent absolute percentage error (MAAPE) calculated as the average of the five repetitions. This type of cross‐validation mimics a problem that plant breeding programs face when they predict genotypes for the next year, given that they have information from the previous years for some genotypes. All analyses were implemented in the R statistical software (R Core Team, 2019); in Supplemental File S1 we provide the key elements for implementing both multi‐trait models.

The main reasons to leave 10% of the observations in the training set are that (1) we should make a reference of the year and site to be predicted; that is, not any unspecified year wanted to be predictive but rather the following year from the previous year where the training was collected; (2) to be able to estimate the effect of environments that is in the model; if one wants to predict 100% of the information of lines of future environment (Year 2) we are unable to estimate the beta coefficients of this environment for the lack of information.

### 2.6 Data availability and software

The following link: http://hdl.handle.net/11529/10548423 contains the Supplemental Information. Supplemental File S1 has the R code for implementing the MRT and BMTME model and Supplemental Table S2 has the average Pearson's correlation (APC) and mean arctangent absolute percentage error (MAAPE) for the testing sets for each data and trait. In addition, the link has the 5 phenotypic and genotypic data from years 2013–2014, 2014–2015, 2015–2016, 2016–2017, 2017–2018.

## 3 RESULTS

### 3.1 Quality trait means

Each year, the lines selected from the yield trial and elite yield trial were fully characterized for their quality characteristics. All the analyzed traits followed an approximately normal distribution (data not shown). On average, wide phenotypic variation could be observed for all the quality tests, especially for mixograph, alveograph and bread loaf volume values (Table 1). However, no great differences could be observed for the average values of the different phenotypes across the trials and data sets (Table 1).

**TABLE 1:** Summary of the wheat quality traits of the lines from the five datasets used for developing and validating the genomic prediction models

	Data 1				Data 2				Data 3				Data 4				Data 5			
Phenotype^a^	YT 2013–2014		EYT 2014–2015		YT 2014–2015		EYT 2015–2016		YT 2015–2016		EYT 2016–2017		YT 2016–2017		EYT 2017–2018		YT 2017–2018		EYT 2018–2019	
	Mean	Stdev	Mean	Stdev	Mean	Stdev	Mean	Stdev	Mean	Stdev	Mean	Stdev	Mean	Stdev	Mean	Stdev	Mean	Stdev	Mean	Stdev
TESTWT	81.8	1.0	80.6	1.1	80.8	1.0	82.9	0.9	82.8	1.0	79.7	1.2	80.1	1.2	80.9	1.1	80.9	1.1	81.1	1.0
TKW	47.8	3.5	45.1	3.0	44.6	3.6	48.9	3.8	48.2	3.7	48.0	3.0	47.4	3.0	48.9	3.4	48.5	3.6	48.9	3.7
GRNHRD^b^	43.5	2.8	46.1	2.2	45.9	2.4	40.2	3.6	42.4	3.5	68.5	5.5	69.6	5.2	67.7	6.1	63.9	9.1	65.4	10.0
GRNPRO	12.2	0.6	12.4	0.6	12.5	0.6	12.4	0.6	12.1	0.6	12.6	0.6	12.8	0.6	13.1	0.7	12.8	0.6	12.4	0.7
FLRPRO	10.7	0.6	10.8	0.6	11.0	0.6	10.8	0.6	10.3	0.6	10.3	0.6	10.4	0.5	10.9	0.6	10.5	0.6	10.7	0.6
FLRSDS	13.7	2.2	13.7	2.2	14.8	2.2	15.7	2.4	15.6	2.2	13.6	1.8	13.1	1.7	13.8	2.2	14.0	2.1	14.1	2.3
MIXTIM	3.0	0.8	3.6	1.0	3.3	0.8	3.0	0.7	3.0	0.6	3.3	0.7	3.2	0.8	2.8	0.7	3.0	0.7	3.1	0.7
MIXTORQ	113.0	28.2	138.2	35.0	126.9	31.6	122.1	29.2	118.6	24.7	130.6	25.9	127.0	30.1	116.1	26.8	123.0	28.1	125.0	28.4
ALVW	253.1	84.2	286.7	91.2	263.7	83.5	289.4	97.8	264.9	79.7	261.2	75.7	253.4	80.4	238.3	76.1	244.3	78.7	229.8	80.3
ALVPL	1.0	0.4	0.8	0.3	0.7	0.2	1.2	0.5	1.1	0.4	1.2	0.5	1.3	0.6	1.3	0.5	1.4	0.5	1.3	0.7
ALVP	–	–	81.9	18.7	75.9	17.8	103.7	28.0	94.9	22.7	99.6	23.5	98.5	24.7	98.3	23.1	100.4	25.1	95.8	28.4
ALVL	–	–	106.1	16.4	107.3	17.3	90.7	17.7	89.9	16.5	83.9	14.2	82.4	16.1	78.7	16.3	78.4	15.5	79.1	18.0
LOFVOL	821.9	58.1	812.0	49.5	821.6	49.5	788.8	54.7	774.6	63.1	744.2	55.1	727.3	55.2	738.1	59.4	733.7	55.0	747.0	45.4

a TESTWT, test weight (kg hl^−1^); TKW, 1000‐kernel weight (g); GRNHRD, grain hardness (PSI and SKCS); GRNPRO, grain protein (%, at 12.5% moisture basis); FLRPRO, flour protein (%, at 14% moisture basis); FLRSDS, flour sodium‐dodecyl‐sulfate sedimentation volume (ml); MIXTIM, mixograph mixing time (min); MP, torque at the integral of the midline peak (‐); ALVW, alveograph (J); ALVPL, ratio between ALVP and ALVL; ALVP, alveograph maximum overpressure (mm); ALVL, alveograph average abscissa of rupture (mm); LOFVOL, pup loaf volume (cm^3^).

b GRNHRD is expressed as Particle Size Index up to years 2015–2016. Starting from the years 2016–2017 it is expressed as Single Kernel Characterization System Hardness Index units.

As expected, when analyzing the correlation among the phenotypes across the different years and data sets, high correlation values were identified between grain and flour protein content. Similarly, relatively high correlation was also identified among the traits indicative of gluten strength such as SDS‐sedimentation volume, mixograph peak time, mixograph torque, alveograph W and alveograph P values. Loaf volume appeared to be relatively highly and positively correlated not only with flour protein content and SDS‐sedimentation, but also with the alveograph parameter L, indicative of dough extensibility (Table 2).

**TABLE 2:** Correlations among the phenotypes across all years and datasets

	TESTWT	TKW	GRNHRD	GRNPRO	FLRPRO	FLRSDS	MIXTIM	MIXTORQ	ALVW	ALVPL	ALVP	ALVL
TKW	0.11											
GRNHRD^a^	−0.46	0.00										
GRNPRO	−0.15	0.06	0.32									
FLRPRO	0.04	−0.05	−0.14	0.78								
FLRSDS	0.18	−0.05	−0.22	0.26	0.40							
MIXTIM	−0.10	−0.20	0.06	−0.06	−0.06	0.34						
MIXTORQ	−0.08	−0.16	0.10	0.04	0.03	0.41	0.97					
ALVW	0.05	−0.12	−0.07	0.18	0.24	0.60	0.81	0.87				
ALVPL	0.01	0.17	0.34	0.01	−0.19	−0.03	0.17	0.25	0.25			
ALVP	0.07	0.11	0.24	0.11	−0.03	0.30	0.50	0.60	0.70	0.83		
ALVL	−0.01	−0.26	−0.40	0.10	0.37	0.39	0.21	0.17	0.24	−0.78	−0.46	
LOFVOL	0.11	−0.23	−0.45	0.22	0.55	0.53	0.25	0.26	0.43	−0.43	−0.08	0.71

†TESTWT, test weight (kg hl^−1^); TKW, 1000‐kernel weight (g); GRNHRD, grain hardness (PSI and SKCS); GRNPRO, grain protein (%, at 12.5% moisture basis); FLRPRO, flour protein (%, at 14% moisture basis); FLRSDS, flour sodium‐dodecyl‐sulfate sedimentation volume (ml); MIXTIM, mixograph mixing time (min); MIXTORQ, torque at the integral of the midline peak (‐); ALVW, alveograph (J); ALVPL, ratio between ALVP and ALVL; ALVP, alveograph maximum overpressure (mm); ALVL, alveograph average abscissa of rupture (mm); LOFVOL, pup loaf volume (cm^3^).

a GRNHRD is expressed as Particle Size Index up to years 2015–2016. Starting from the years 2016–2017 it is expressed as Single Kernel Characterization System Hardness Index units.

### 3.2 Genomic prediction

The results of the genomic prediction analysis are given in five sections, one for each data set created, and the prediction performance of each is reported in terms of average Pearson's correlation and MAAPE. The results used to generate all five data figures are given in Supplemental Table S2.

### 3.3 Data 1 (lines from year 2013–2014)

**FIGURE 1:**
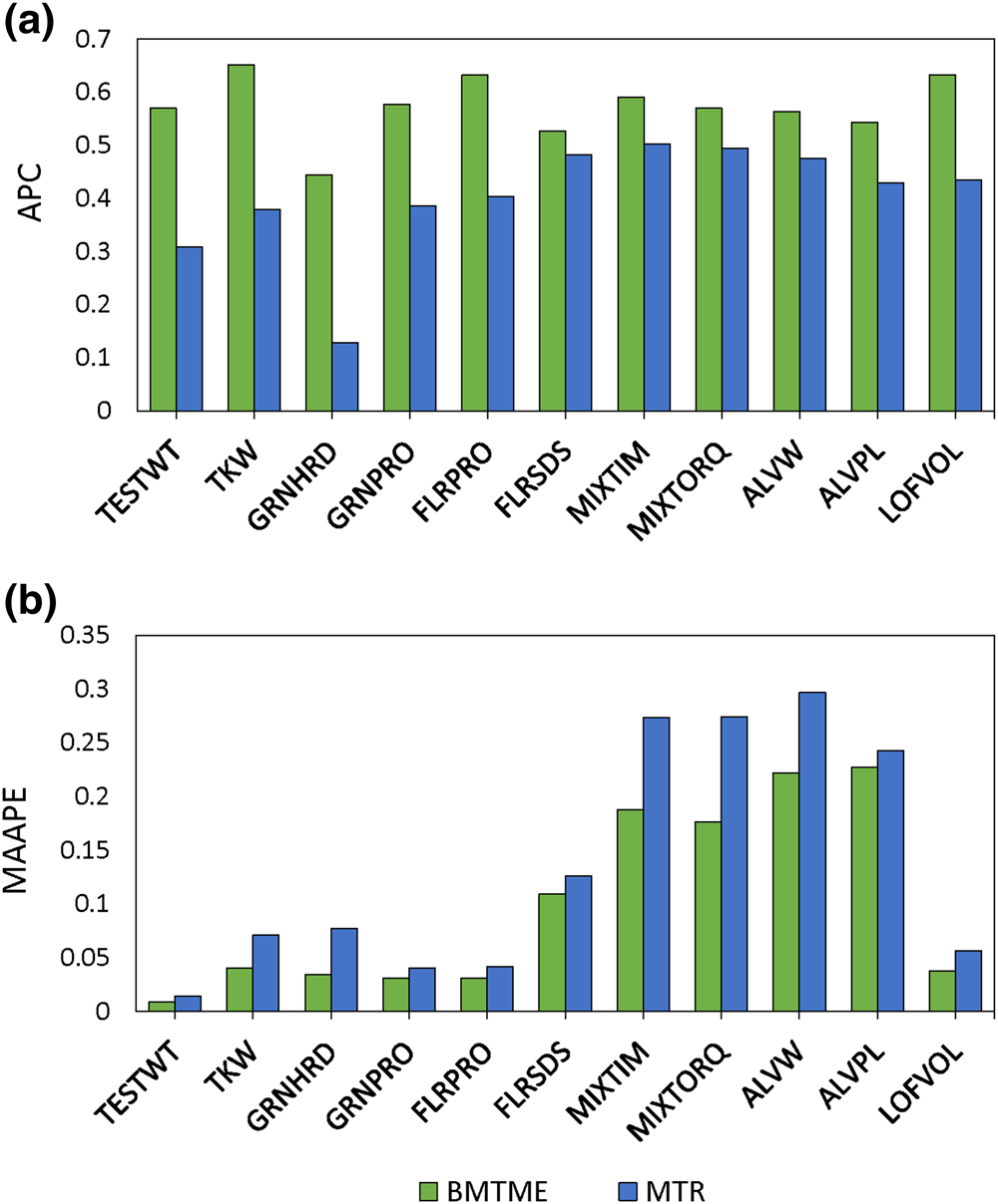
(a) Average Pearson's correlation (APC) and (b) Mean arctangent absolute percentage error (MAAPE) of testing sets for the five partitions for Data 1. The testing set is composed of 90% of the lines of the second year (testing), while the training set comprises all the information of the first year and 10% of the same lines evaluated in the second year

Figure 1a shows the prediction performance of all traits under study under both models (BMTME and MTR). This figure indicates that the best predictions were obtained with the BMTME model and that the largest difference between the BMTME and MTR (multi‐trait ridge regression) was observed for GRNHRD where the BMTME model outperformed the MTR model by 147.6%. Under the BMTME model, the lowest prediction was 0.444 (trait GRNHRD), whereas the highest prediction was 0.652 (trait TKW). Similar to the BMTME model, GRNHRD was associated with the lowest prediction also under the MTR model (0.128). The highest prediction accuracy using MTR was associated with MIXTIM (0.503). When comparing the two models for the MAAPE (Figure 1b), the BMTME model again outperformed the MTR model for all traits. In this case, the largest difference in terms of prediction between both models was observed for traits MIXTIM and MIXTORQ, where the BMTME model outperformed the MTR model by 45.7% and 55.4%, respectively. Under the BMTME model, the worst prediction was for trait ALVPL with a MAAPE value of 0.227, while the best was for trait TESTWT with a MAAPE value of 0.009. On the other hand, under the MTR model, the best predictions were for trait TESTWT (MAAPE = 0.014) and the worst for trait ALVW (MAAPE = 0.297).

### 3.4 Data 2 (lines from year 2014–2015)

**FIGURE 2:**
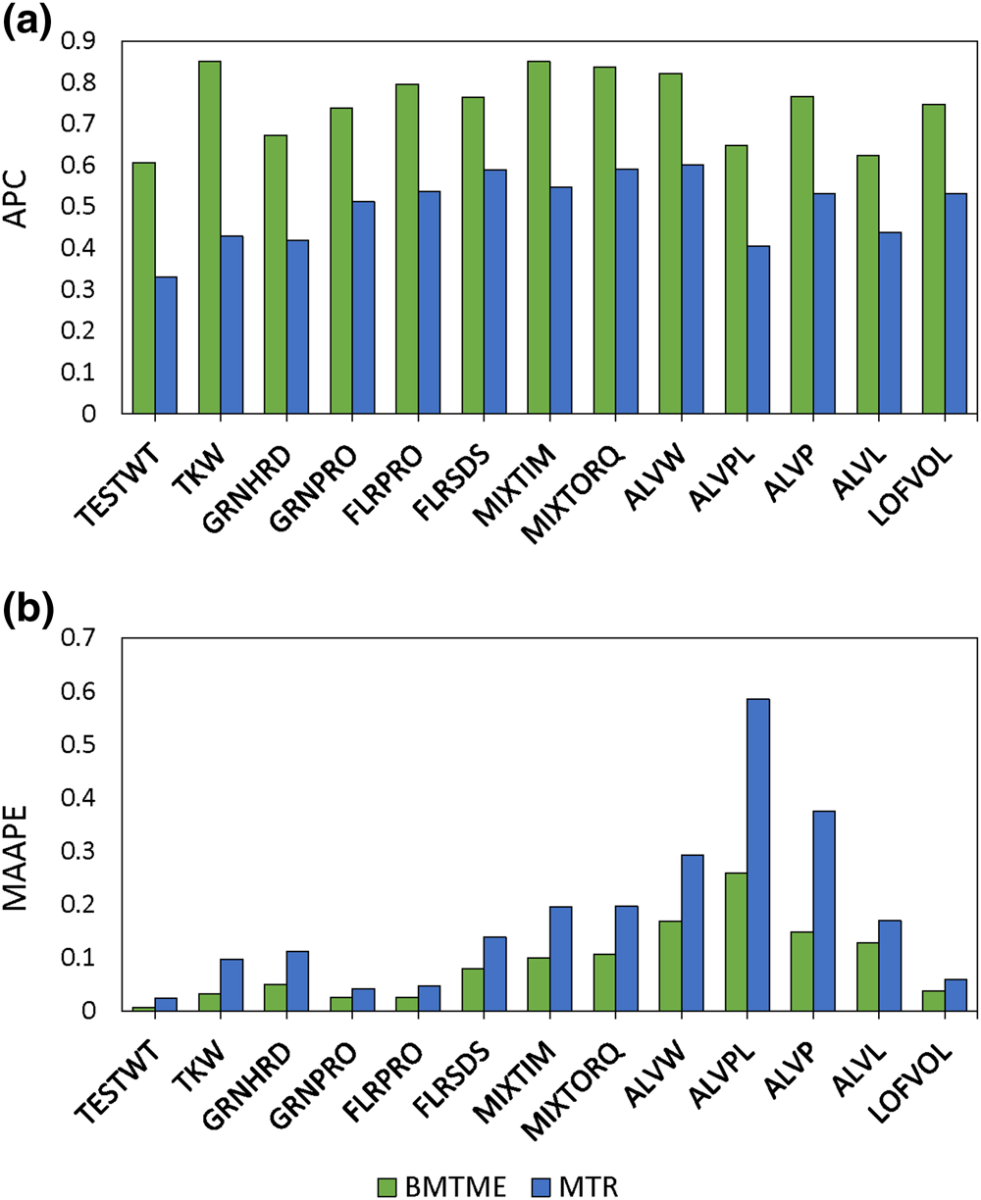
(a) Average Pearson's correlation (APC) and (b) Mean arctangent absolute percentage error (MAAPE) of testing sets for the five partitions for Data 2. The testing set is composed of 90% of the lines of the second year (testing), while the training set is composed of all the information of the first year and 10% of the lines of the second year

Using the lines from Data 2, the BMTME model overall appeared to be better than the multi‐trait ridge regression model. As shown in Figure 2a, the best predictions were obtained with the BMTME model for all traits and the largest difference between both models (BMTME and MTR) was obtained for TKW where the BMTME model outperformed the MTR model by 98.5%. Using the BMTME model, the lowest prediction accuracy was for TESTWT, which was equal to 0.607, whereas the best prediction was for TKW (0.851). Interestingly, TESTWT was associated with the lowest prediction accuracy also using the MTR model (0.331), whereas the alveograph parameter ALVW reported the best prediction (0.601). When analyzing the MAAPE (Figure 2b **),** the best predictions were again obtained under the BMTME model for all 13 traits. In this case, the largest difference between the two models was for ALVPL, where the MTR model appeared to be worse than the BMTME model by 125.4%. Under the BMTME model, the worst prediction was for ALVPL with a MAAPE value of 0.259, whereas the best was for trait TESTWT with a MAAPE value of 0.007. On the other hand, under the MTR model the best predictions were for trait TESTWT (MAAPE = 0.0237) and the worst for trait ALVW (MAAPE = 0.585).

### 3.5 Data 3 (lines from years 2015–2016)

**FIGURE 3:**
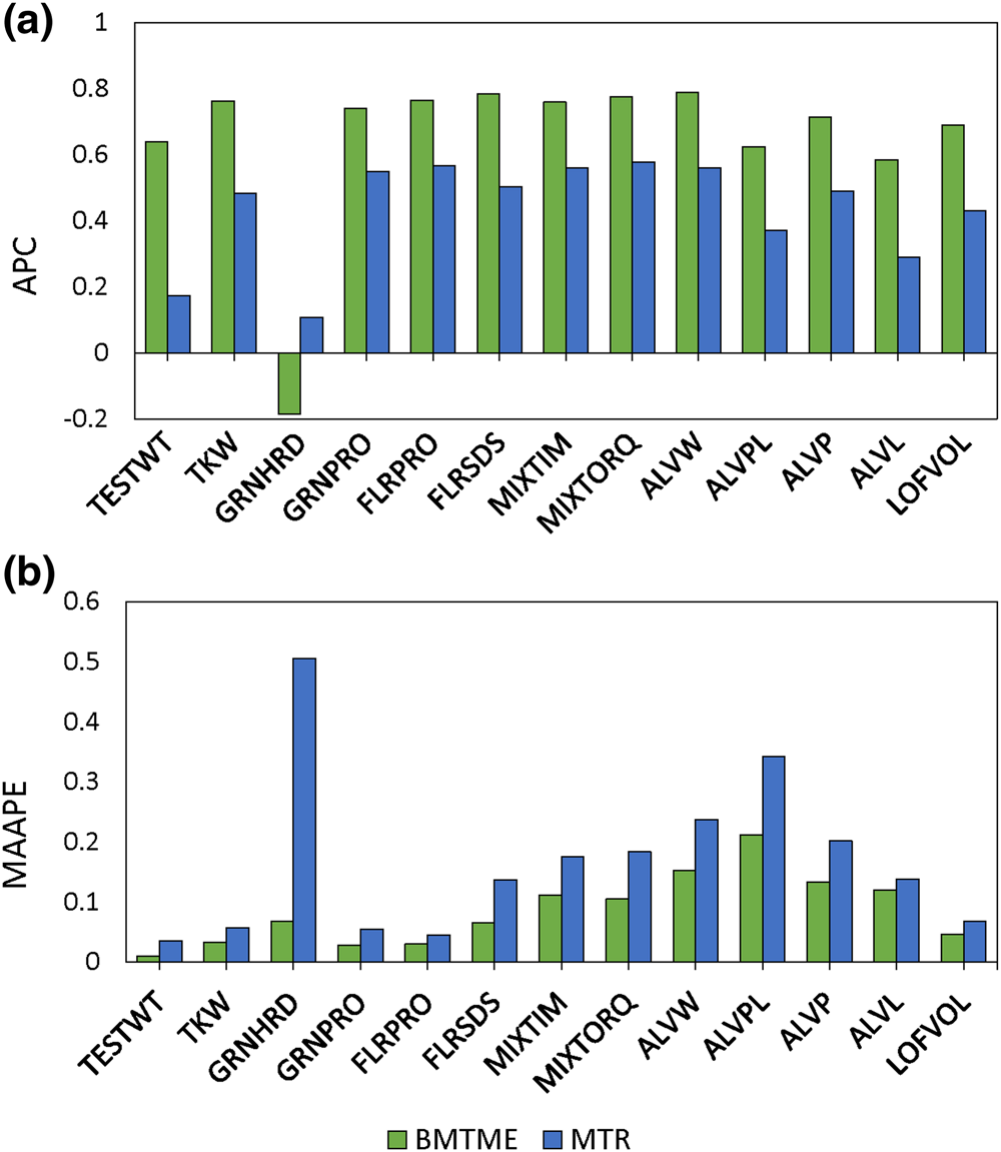
(a) Average Pearson's correlation (APC) and (b) Mean arctangent absolute percentage error (MAAPE) of the testing sets for the five partitions for Data 3. The testing set is composed of 90% of the lines of the second year (testing), while the training set is composed of all the information of the first year and 10% of the lines of the second year

When using the lines from Data 3, the BMTME model also appeared to outperform the MTR model. In fact, for all 13 traits, the BMTME model gave greater prediction accuracies compared to the MTR model (Figure 3a). Among the traits, the greatest difference in prediction accuracy was for TESTWT, where the prediction of the BMTME model appeared to be 267.7% better than the one obtained using the MTR model. Using the BMTME model, the best prediction was for trait ALVW (0.788), whereas the lowest was associated with the alveograph parameter ALVL (0.584). Under the MTR model, the best prediction was for trait MIXTORQ (APC = 0.577), whereas the worst was for TESTWT, which was associated with an average Pearson's correlation equal to 0.174. As expected, the APC values associated with grain hardness (GRNHRD) obtained with both models were extremely low (BMTME, −0.186; MTR, 0.108). These results may be explained by the fact that the grain hardness of the lines grown in 2015–2016 (training population) and the lines grown in 2016–2017 (testing population) was analyzed using two different methods. When analyzing using the MAAPE metric (Figure 3b), the BMTME model appeared to outperform the MTR model for all traits. The worst performance of the MTR model was for trait GRNHRD and the BMTME model was superior to the MTR model by 652.9%. The worst and best predictions under the BMTME model were 0.212 and 0.009, respectively, corresponding to traits ALVPL and TESTWT. Without taking into consideration grain hardness, under the MTR model the worst prediction was for ALVPL, which was associated with a MAAPE = 0.343. The best prediction was observed for TESTWT, which was associated with a MAAPE = 0.035.

### 3.6 Data 4 (lines from years 2016–2017)

**FIGURE 4:**
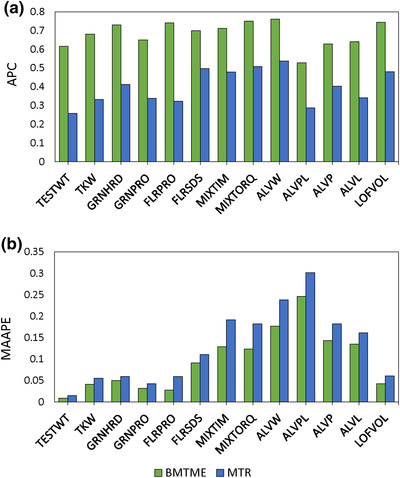
(a) Average Pearson's correlation and (b) Mean arctangent absolute percentage error (MAAPE) of the testing sets for the five partitions for Data 4. The testing set is composed of 90% of the lines of the second year (testing), while the training set is composed of all the information of the first year and 10% of the lines of the second year

The results obtained using the three previous datasets were confirmed when using the lines from the fourth dataset. In this case, the BMTME model also outperformed the MTR model for all traits, as indicated by both the APC and MAAPE values (Figure 4). Specifically, the BMTME model was associated with greater APC values for all traits (Figure 4a) and the largest gain (129.516%) of the BMTME model over the MTR model was observed for FLRPRO. The best prediction under the BMTME model was for ALVW, which was associated with an average Pearson's correlation equal to 0.760. In contrast, the alveograph trait ALVPL obtained the worst prediction performance with an average Pearson's correlation equal to 0.528. Under the MTR model, trait ALVW was also the one better predicted (APC = 0.538), whereas the worst correlation value was for trait TESTWT (0.259). The BMTME model outperformed the MTR model in all traits also under the MAAPE metric (Figure 4b), and specifically for the mixograph mixing time (MIXTIM), the BMTME model was better than the MTR model by 48.5%. However, interestingly, using both the BMTME and the MTR model, the worst MAAPE values were associated with ALVPL (0.246 and 0.301, respectively), whereas the best MAAPE values were associated with TESTWT (0.009 and 0.015, respectively).

### 3.7 Data 5 (lines from years 2017–2018)

**FIGURE 5:**
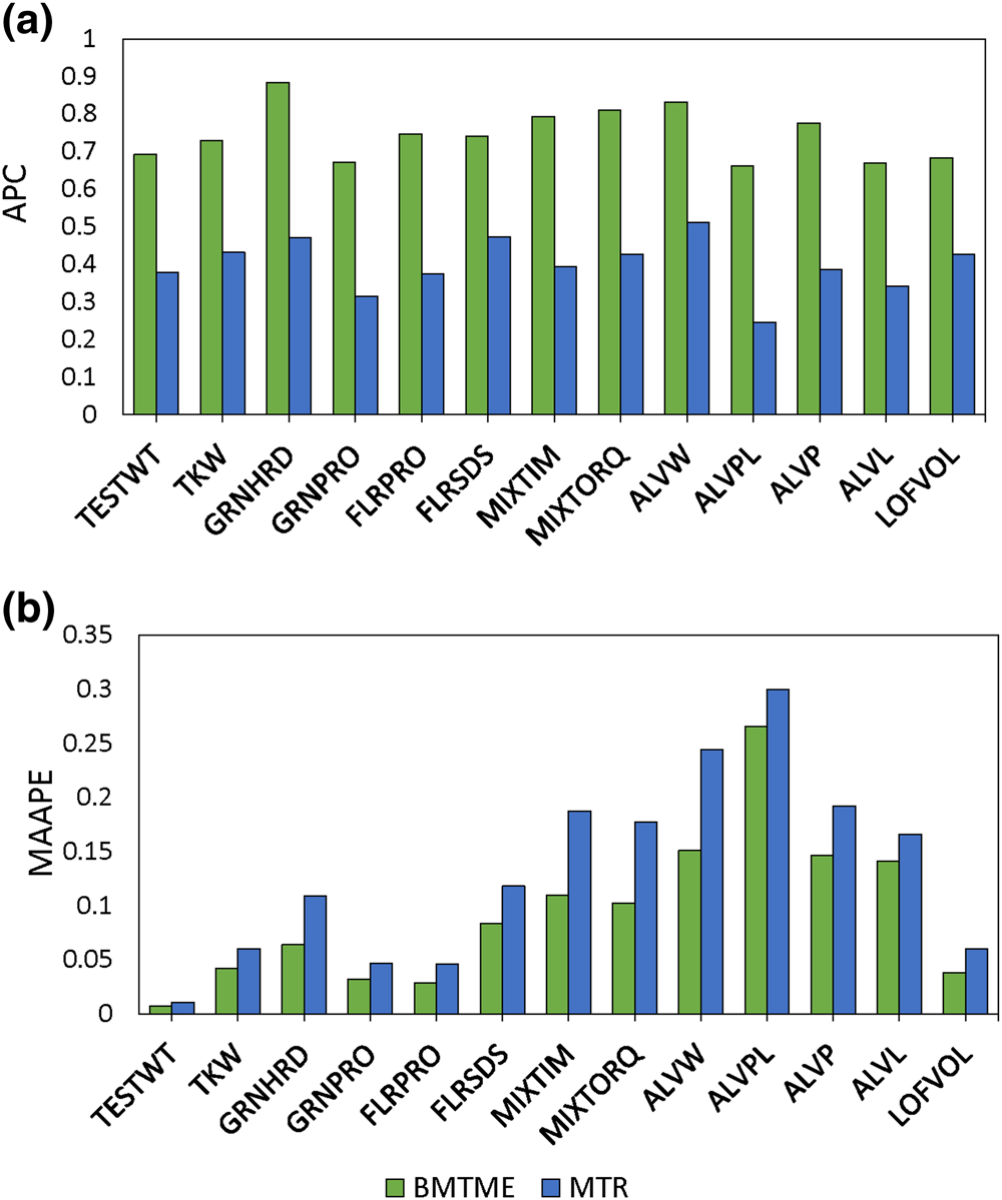
(a) Average Pearson's correlation (APC) and (b) Mean arctangent absolute percentage error (MAAPE) of the testing sets for the five partitions for Data 5. The testing set is composed of 90% of the lines of the second year (testing), while the training set is composed of all the information of the first year and 10% of the lines of the second year

As for the other datasets, using the lines from the 2017–2018 cycle (Data 5), the BMTME model appeared to be superior to the MTR model. As described in Figure 5a, the BMTME model outperformed the MTR model in all traits in terms of average Pearson's correlation. Specifically, the largest difference between the BMTME and MTR models was for trait ALVPL, where the BMTME outperformed the MTR model by 169.6%. The minimum average Pearson's correlation under the BMTME model was for trait ALVPL (0.661), whereas the maximum APC value was for GRNHRD (0.884). In contrast, under the MTR model, the minimum average Pearson's correlation was 0.245 associated with the alveograph parameter ALVPL, whereas the maximum value was 0.510 associated with the other alveograph parameter ALVW. When analyzing the MAAPE, the BMTME model again outperformed the MTR model for all traits (Figure 5b). In this case, the largest difference between the two models was for trait ALVW for which the BMTME model outperformed the MTR model by 61.8%. Under the BMTME model, the best and worst MAAPE values were associated with traits TESTWT and ALVPL (0.007 and 0.265, respectively). Similarly, when using the MTR model, the best and worst percentage errors were associated with TESTWT (0.010) and ALVPL (0.300).

## 4 DISCUSSION

In this paper, we evaluated the feasibility of implementing genomic selection for the selection of wheat processing and end‐use quality traits. Genomic selection applied to wheat quality would in fact be very useful, since wheat quality evaluation usually happens only in the last stages of the breeding cycle and is expensive and time‐consuming. Using two statistical models (BMTME and MTR) to predict 13 different wheat quality traits, we showed that it is possible to accurately predict the quality of 90% of the lines grown in the second year using as training population the lines grown in the first year and 10% of the lines grown in the second year. As expected, the prediction correlation values and accuracies differed depending on the statistical model, the trait to be predicted and the dataset used to train the statistical models.

When performing genomic selection, multi‐trait analysis is typically preferred over univariate analysis when the degree of correlation between traits is moderate or high (Calus & Veerkamp, 2011; He, Kuhn, & Parida, 2016; Jia & Jannink, 2012; Jiang et al., 2015; Montesinos‐López et al., 2016; Schulthess, Zhao, Longin, & Reif, 2017). For this reason, the BMTME generalizes conventional multi‐trait analysis (which takes into account the correlation between traits) to also take into account the correlation between environments. In the context of deep learning, there are applications of multi‐trait deep learning for genomic selection such as those published by Montesinos‐López et al. (2018) and Montesinos‐López et al. (2019c, 2019d), in which a small gain in terms of prediction performance is obtained with regard to univariate deep learning prediction.

Of the two statistical models used in the present study, the BMTME proved to be consistently better compared to the MTR model (implemented with *glmnet*). The large difference in terms of prediction performance between the two models can be mainly attributed to three factors: (1) the BMTME model provides a separate penalization for environment, for the genotypes (lines) and for genotype × environment interaction, whereas the MTR model only provides one penalization for the whole set of independent variables (environment, genotypes and genotype × environment interaction); (2) the training process used for the two models is different, since the BMTME model was trained using both the training set (with genotype and phenotypic information) and the genotypic information of the testing set, whereas the MTR model was trained using only the information of the training set (with genotype and phenotypic information); (3) the BMTME, being a Bayesian model, used weak prior information during the training process, which helped to improve the prediction process, whereas the MTR model did not use any prior information. However, it must be noted that the BMTME is quite costly in terms of implementation time compared to the MTR, which is very fast. For this reason, more research is necessary to be able to reduce the implementation time of the BMTME model, which will help to routinely use this method in GS programs.

Regardless of the model adopted for prediction, in the present study the mean arctangent absolute percentage error (MAAPE) was also used as a method to evaluate the models’ prediction accuracy. The MAAPE has several advantages over similar methods. It is in fact: (1) easy to interpret (the lower the value, the better the prediction performance); (2) it can be used to compare the prediction accuracy among traits measured in different scales, which is not possible using the mean square error of prediction; (3) and finally, unlike Pearson's correlation, it does not require calculating other metrics such as the intercept (the best: close to zero) and slope (the best: close to 1) to make sure that the results obtained are not misleading. For these reasons, we encourage using the MAAPE metrics to evaluate the prediction accuracy of continuous and count traits in plant breeding.

When analyzing the prediction accuracies associated with each quality trait, we found that the results obtained in the present study exceeded (Battenfield et al., 2016; Kristensen et al., 2019) or were comparable to (Haile et al., 2018; Juliana et al., 2019) the results reported in previous studies.

With respect to the predicted quality traits, in general, the traits associated with grain morphological characteristics (TESTWT and TKW) and with protein content (GRNPRO and FLRPRO) under both the BMTME and MTR models and across the datasets, had relatively high Pearson's correlation values associated with low MAAPE values (high prediction accuracy). Specifically, *r* values as high as 0.69 and 0.85 were identified for TESTWT weight and TKW, which appeared to be greater than the results reported by Battenfield et al. (2016) (TESTWT max *r* = 0.34 and TKW max *r* = 0.48) and Juliana et al. (2019) (TESTWT *r *= 0.45–0.52 and TKW *r* = 0.62–0.56) possibly because of both the different statistical model used and the population set analyzed. In all cases, TKW appeared to be more highly predictable than TESTWT.

Similarly, protein content in both flour and grain could be effectively predicted with relatively high prediction accuracies (*r* from 0.57 to 0.79). These results are comparable to the ones reported by Haile et al. (2018) in a durum wheat breeding population, and by Juliana et al. (2019), but higher than the results reported by Battenfield et al. (2016). Protein content is a very important trait that is necessary to better understand and interpret the results from the other quality tests. However, even though protein content is highly influenced by the environment (Mahjourimajd et al., 2016), the prediction accuracies obtained in the present study would suggest that it is possible to accurately predict protein content, thus improving the overall selection for wheat quality.

In contrast, a marked difference in GRNHRD predictive ability could be observed over the datasets and the years (Data 1 *r* = 0.44 vs Data 5 *r* = 0.88). As reported in the previous sections, the lines of the first two datasets were analyzed for grain hardness using NIR, whereas the hardness of the lines in the last two datasets was analyzed using the SKCS. These results confirm the superiority of the SKCS for analyzing grain hardness but also stress the importance of an accurate phenotypic characterization to obtain an efficient and reliable prediction. Using datasets 4 and 5, Pearson's correlation values for grain hardness were much greater compared to the results reported by Battenfield et al. (2016) (*r* = 0.32, year 2015) and Juliana et al. (2019) (*r* = 0.45–0.55), where this trait was also measured using NIR.

The traits indicating gluten strength, such as SDS‐sedimentation volume, the two mixograph parameters MIXTIM and MIXTORQ and the alveograph parameters ALVW and ALVP, were all associated with relatively high average Pearson's correlation values but also with higher MAAPE values when compared to the other traits. Similar results were reported by Battenfield et al. (2016), Kristensen et al. (2018), 2019) and Juliana et al. (2019), where among the quality traits analyzed, the ones associated with gluten strength appeared to be associated with high Pearson's correlation values compared to the other analyzed traits. As reported in the literature, gluten strength is, in fact, a relatively highly heritable trait (*h^2^* > 0.6, Battenfield et al., 2016; Kristensen et al., 2019) being influenced mainly by the variation of the gluten‐forming proteins (Shewry, Halford, & Lafiandra, 2003). In contrast, the two alveograph parameters ALVL and ALVPL indicative of dough extensibility and of the balance between dough extensibility and strength, respectively, were more difficult to predict and were also associated with greater MAAPE values (lower prediction accuracy). Similar results were reported in the study of Battenfield et al. (2016) and Juliana et al. (2019), where the parameter ALVPL was associated with the lowest correlation values, and by Kristensen et al. (2019), where ALVL was associated with the lowest predictive ability among the alveograph parameters. Together, the results reported here and in previous studies suggest that dough extensibility, as expressed by the alveograph parameters ALVL and ALVPL, is hard to predict and highly affected by several factors other than genetics. Interestingly, loaf volume, which is a very important but also expensive test for the characterization of the end‐use quality of a bread wheat line, was predicted quite efficiently using the BMTME model (APC ranging from 0.63 to 0.74), as also reported by Juliana et al. (2019).

Given the results obtained in the present study, it appears to be feasible to implement genomic selection in the breeding cycle for predicting the majority of the wheat quality parameters. Following the scheme adopted here, it would be theoretically possible to reduce by 90% the number of lines to be analyzed for quality in the second year, thus dramatically reducing the cost and the time required for the wheat quality analyses. The cost of genotyping each sample through GBS is in fact around 10–20 USD per sample, which is approximately ten times lower than the cost of a full quality characterization of a single wheat line (around 100 to 200 USD per sample). Also in terms of efficiency, genotyping appears to be preferable compared to phenotyping for wheat quality since it can be performed at the early stages of the plant cycle and on a greater number of samples in a shorter time. At CIMMYT, it is in fact possible to analyze through GBS around 9,000 wheat lines in a period of three months, whereas in the same time no more than 2,000 lines could be completely characterized for wheat quality.

However, it is important to highlight that successful prediction is inextricably associated with robust and reliable phenotypic characterization, which by no means can be substituted or eliminated but could be limited to a lower number of samples. As also reported by Battenfield et al. (2016), genomic selection applied to wheat quality cannot be considered an absolute replacement for the quality characterization of a wheat line, but rather a tool for improving selection efficiency throughout the breeding cycle. In this respect, genomic prediction could have a great potential for backward selection. For example, the quality data generated each year for a selected number of wheat lines could be used to predict the quality of the rest of the lines grown in the same year but not included in the wheat quality analysis for different reasons (limited budget, limited quantity of seed etc.). In this way, decisions regarding the advancement of different wheat lines along the breeding cycle could be made more consciously and the genetic potential of a breeding program for wheat quality could be better exploited.

## 5 CONCLUSIONS

In this paper we evaluated the prediction performance of two multi‐trait models (BMTME and MTR) using 13 quality traits of wheat. We found that it is feasible to implement genomic selection for identifying the lines to be advanced in the breeding cycle using the data from the current year, since moderate to good prediction performances were identified for most traits. We also found that the best predictions were obtained under the BMTME model, which is expected when the correlation among traits and among environments is moderate to large. The data considered in this study is from optimum environments; therefore, further research is needed to assess the genomic‐enabled prediction accuracy of these wheat quality traits under differential environmental conditions as drought, heat, late heat, etc. Also, in this study we predicted the quality traits across years rather than across generations. Application of genomic selection for the prediction of the quality of the first‐year untested material could be at least twice more efficient compared to the selection efficiency obtained here, both in terms of cost and genetic gain. More studies will be performed to verify the improvement in the selection efficiency obtained from the implementation of GS at different stages of the breeding process.

## CONFLICT OF INTEREST DISCLOSURE

The authors declare that there is no conflict of interest.
